# Epstein-Barr Virus-Induced Gene 3 (EBI3): A Novel Diagnosis Marker in Burkitt Lymphoma and Diffuse Large B-Cell Lymphoma

**DOI:** 10.1371/journal.pone.0024617

**Published:** 2011-09-08

**Authors:** Julie Gonin, Frédérique Larousserie, Christian Bastard, Jean-Michel Picquenot, Jérôme Couturier, Isabelle Radford-Weiss, Céline Dietrich, Nicole Brousse, Marie-Cécile Vacher-Lavenu, Odile Devergne

**Affiliations:** 1 CNRS UMR 8147, Université Paris Descartes, Sorbonne Paris Cité, Paris, France; 2 Service d'Anatomie Pathologique, AP-HP, Hôpital Cochin, Paris, France; 3 Laboratoire de Génétique Oncologique, Centre Henri Becquerel, Rouen, France; 4 Laboratoire d'Anatomie Pathologique, Centre Henri Becquerel, Rouen, France; 5 Service de Génétique Oncologique, Institut Curie, Paris, France; 6 Laboratoire de Cytogénétique, AP-HP, Hôpital Necker, Paris, France; 7 Service d'Anatomie Pathologique, AP-HP, Hôpital Necker, Paris, France; Mayo Clinic, United States of America

## Abstract

The distinction between Burkitt lymphoma (BL) and diffuse large B-cell lymphoma (DLBCL), two types of mature aggressive B-cell lymphomas that require distinct treatments, can be difficult because of forms showing features intermediate between DLBCL and BL (here called BL/DLBCL). They can be discriminated by the presence of *c-myc* translocations characteristic of BL. However, these are not exclusive of BL and when present in DLBCL are associated with lower survival. In this study, we show that Epstein-Barr virus-induced gene 3 (EBI3) is differentially expressed among BL and DLBCL. Analysis of gene expression data from 502 cases of aggressive mature B-cell lymphomas available on Gene Expression Omnibus and immunohistochemical analysis of 184 cases of BL, BL/DLBCL or DLBCL, showed that EBI3 was not expressed in EBV-positive or -negative BL cases, whereas it was expressed by over 30% of tumoral cells in nearly 80% of DLBCL cases, independently of their subtypes. In addition, we show that c-myc overexpression represses *EBI3* expression, and that DLBCL or BL/DLBCL cases with *c-myc* translocations have lower expression of EBI3. Thus, EBI3 immunohistochemistry could be useful to discriminate BL from DLBCL, and to identify cases of BL/DLBCL or DLBCL with potential *c-myc* translocations.

## Introduction

Burkitt's lymphoma (BL) and diffuse large B-cell lymphoma (DLBCL) are mature aggressive B-cell lymphomas whose distinction can be tricky. Their diagnosis relies on the patient's clinical data and on specific features of the lymphoma including morphology, immunophenotype, and cytogenetic abnormalities. BL is a homogenous group characterized by c-myc overexpression as a result of *c-myc* gene translocation, and consequently increased proliferation. This translocation juxtaposes the locus of *c-myc* gene to one of the Ig loci (heavy chain, lambda or kappa light chains) [1]. In contrast, DLBCL encompasses a heterogeneous group of B-cell lymphomas with clinical, morphological, immunohistochemical and molecular subtypes [2], [3]. Accurate diagnosis of BL and DLBCL is essential because adequate chemotherapy regimen differs between both types of lymphomas. BL is cured by high intensity chemotherapy, whereas DLBCL is usually treated by lower-dose chemotherapy regimens: cyclophosphamide, doxorubicin, vincristine, and prednisone (CHOP), in association with rituximab anti-CD20 antibody (R-CHOP) [1], [3]–[5].

Although *Ig-myc* translocation is the hallmark of BL, *c-myc* translocations are also found in other lymphomas. In particular, they are found in a subset (5 to 15%) of DLBCL and in a high proportion of lymphomas that are borderline between BL and DLBCL and were previously called « atypical BL » or « BL-like lymphoma ». These latter lymphomas are now categorized as « B-cell lymphomas, unclassifiable, with features intermediate between DLBCL and BL » [6], and will be referred to as BL/DLBCL in this study. In BL/DLBCL and DLBCL, *c-myc* translocations often involve non-Ig partners and are associated with a complex caryotype. Several studies have shown that these cases represent aggressive forms with poor prognosis [7]–[12] and the most appropriate treatments remain a matter of debate. In particular, a recent study showed that, among DLBCL patients treated with R-CHOP chemotherapy, those having *c-myc* gene rearrangements had an inferior prognosis compared to those without *c-myc* translocations, and it was suggested that treatment regimens similar to those used in BL would be more appropriate for these cases [11]. These observations highlighted the importance of identifying cases of DLBCL with *c-myc* translocations. However, cytogenetic studies are not systematically performed.

In previous immunohistochemical studies, we showed that Epstein-Barr virus (EBV)-induced gene 3 (EBI3), a molecule related to the p40 subunit of interleukin (IL)-12 [13], exhibited a restricted expression profile among B-cell lymphomas [14], [15]. We found that EBI3, which was originally characterized as a gene induced in EBV-transformed B cells by the viral oncogene LMP1 [13], [16], was also expressed in certain non-EBV-associated B-cell lymphomas such as DLBCL. Indeed, EBI3 was found to be expressed by tumoral cells in 18/22 cases of DLBCL [15], whereas it was not expressed in 6/6 cases of EBV-positive BL [17], consistent with the absence of LMP1 expression in EBV-associated BL. Subsequently, a study of gene profiling by Dave *et al* [18] showed that *EBI3* was among the NF-κB regulated genes that were selectively overexpressed in DLBCL compared to BL.

These observations prompted us to further analyze the expression of EBI3 in large series of BL and DLBCL to clearly establish its differential expression profile among both types of lymphomas, and the usefulness of EBI3 immunohistochemistry for their differential diagnosis. In addition, we investigated whether EBI3 immunohistochemistry could be used as a tool to identify cases with potential *c-myc* gene rearrangements among BL/DLBCL and DLBCL.

## Methods

### Lymphomas

Formalin-fixed paraffin-embedded tumor biopsies from 184 cases of mature aggressive B-cell lymphomas diagnosed between 1987 and 2009 at Necker Hospital (Paris), Cochin Hospital (Paris) or the Henri Becquerel Cancer Center (Rouen) were included in this study. All cases were reviewed anew and classified according to the World Health Organization 2008 lymphoma classification [6]. Tumors classified as BL (n = 23) had both morphologic features and an immunophenotype typical of BL (positivity for both CD10 and Bcl6, and proliferation index measured by Ki67 positivity >90%). EBV (EBER probe) was detected in 4/18 cases, and all but one of cases were negative for Bcl2. Cases of DLBCL (n = 138) were classified into germinal center (GC) B-cell-like (GCB) or non-GCB types, based on the differential immunodetection of CD10, Bcl6, and MUM1, as previously described [15], [19]. For these markers, cases were considered positive if ≥30% of tumoral cells stained positive. This category contains only one case of primary mediastinal B-cell lymphoma (PMBL). The BL/DLBCL category comprised cases (n = 23) with morphological features that were intermediate between DLBCL and BL, or cases with morphological features of BL but a deviant immunophenotype based on Ki67, Bcl6, and CD10 staining. A case of follicular lymphoma and its transformed counterpart were also analyzed. In some cases (n = 16), frozen tissues were analyzed.

Translocations for *c-myc* were identified by conventional cytogenetics using RHG-banding techniques on 24-hour cultures of cells isolated from fresh tissue biopsies (103 cases), by fluorescence *in situ* hybridization (FISH) using MYC dual color break apart rearrangement probe (Vysis) performed on frozen or paraffin-embedded tissues (13 cases) or both techniques (12 cases). Cytogenetic data were obtained in 17/23 cases (74%) of BL, 88/138 cases (64%) of DLBCL and in all BL/DLBCL cases. *C-myc* translocations were detected in 17/17 of BL cases, 12/88 (14%) of DLBCL cases and 19/23 (83%) of BL/DLBCL cases. The main characteristics of the lymphomas are summarized on Table 1.

**Table 1:** Characteristics of mature aggressive B-cell lymphomas analyzed by immunohistochemistry.

				*c-myc* translocation		
	No. of			No. of cases		
Diagnosis	cases	Subtype		analyzed	present	absent
BL	23	GCB	23 (100%)	17	17 (100%)	0
BL/DLBCL	23	GCB	21 (91%)	21	17 (81%)	4 (19%)
		non-GCB	0			
		not determined	2 (9%)	2	2	0
DLBCL	138	GCB	49 (35%)	33	7 (21%)	26 (79%)
		non-GCB	85 (62%)	53	4 (8%)	49 (92%)
		not determined	4 (3%)	2	1	1

All tissues were collected for histological examination and diagnosis purpose and were studied in accordance with the French ethical laws for studies on human tissues and with the Declaration of Helsinki. This study was approved by the institutional review board of the hospitals and cancer center that provided tissue samples and were involved in the study.

### EBI3 immunohistochemistry

EBI3 immunostaining was performed on formalin-fixed paraffin-embedded tissue sections. Sections were dewaxed, rehydrated and subjected to antigen retrieval by heat pretratment using citrate buffer. Endogeneous peroxidase activity was quenched with a peroxide-methanol buffer. EBI3 was detected using 2G4H6 mouse monoclonal antibody (IgG2a), the characterization and specificity of which had been previously reported [20], at 2–4 µg/ml. Specificity of the staining was controlled by testing in parallel an isotype-matched control mAb (RPC5, IgG2a, Cappel Durham). Binding of the primary antibody was detected by an indirect avidin-biotin peroxidase technique (BioGenex) and DAB as chromogen. Sections were counterstained with Mayer hematoxylin. Images were captured on a NanoZoomer 2.0-RS slide scanner (Hamamatsu Corporation) and processed with NDP Viewer. The percentage of EBI3-positive tumoral cells was determined in a blinded fashion (without knowledge of *c-myc* status) by two pathologists (JG and FL), independently.

### Plasmids, cell line and transfection

An EcoRI fragment encoding an inducible c-myc-estrogen receptor (Myc-ER) fusion protein was inserted in the EcoRI site of pSG5 to construct pSG5-c-myc-ER plasmid. The green fluorescent protein (GFP) expression vector, pEGFP-C1, was obtained from Clontech.

Karpas 1106, a DLBCL cell line derived from a patient with a mediastinal form of DLBCL, was purchased from the Deutsche Sammlung von Mikroorganismen und Zemmkulturen Gmbh, Germany. It is negative for EBV and does not carry *c-myc* translocation. It was cultured in RPMI 1640 medium supplemented with 20% fetal calf serum, L-glutamine and antibiotics.

For transient expression, Karpas 1106 cells (5×10^6^ cells per cuvette) were electroporated with pSG5 control plasmid or pSG5-c-myc-ER together with GFP expression vector on a BioradGene Pulser Xcell electroporator at 250 V and 500 µF in 200 µl of RPMI medium containing 10% fetal calf serum. Two hours after electroporation (or at later time points in some cases), 4-hydroxytamoxifen (4-OHT, Sigma-Aldrich) was added at a final concentration of 200 nM - unless otherwise specified - to activate c-myc-ER fusion protein. Although we did not observe any significant effect of 4-OHT on *EBI3* expression in Karpas 1106 cells (not shown), 4-OHT was added to both pSG5 control and pSG5-c-myc-ER transfected cells to exclude a non-specific effect. Sixteen to 40 hours after 4-OHT addition, GFP-positive cells were isolated by electronic cell sorting on a FACSAria cytometer (IRNEM cell sorting facility). GFP-positive cells from c-myc-ER transfectants were verified to express c-myc-ER fusion protein by western blot (not shown).

### Real time quantitative PCR (RTqPCR)

Total RNA was isolated from purified GFP-positive transfected cells or from 20 µm frozen tissues sections by TRIzol extraction followed by DNAse I digestion, or by using the RNeasy Plus Micro kit (Qiagen). RNA was reverse transcribed into cDNA using M-MLV reverse transcriptase, and oligo(dT) or random hexamer primers (all reagents from Invitrogen). RTqPCR for *EBI3* and *c-myc* was performed using Taqman Universal PCR Master mix and TaqMan® gene expression assays. For each sample, triplicate reactions were run for 40 cycles on Step One Plus thermal cycler (Applied Biosystems). Levels of target mRNA were normalized relative to levels of ß2-microglobulin mRNA, and relative mRNA expression was calculated using the comparative cycle threshold method.

### Statistical analysis

Statistical analysis was performed by Mann-Whitney or Student' t test. Correlation was analyzed by Spearman test. A p value<0.05 was considered to indicate statistical significance.

## Results

### BL and DLBCL, defined by molecular profiling, are characterized by differential expression of *EBI3* gene

**Figure 1:**
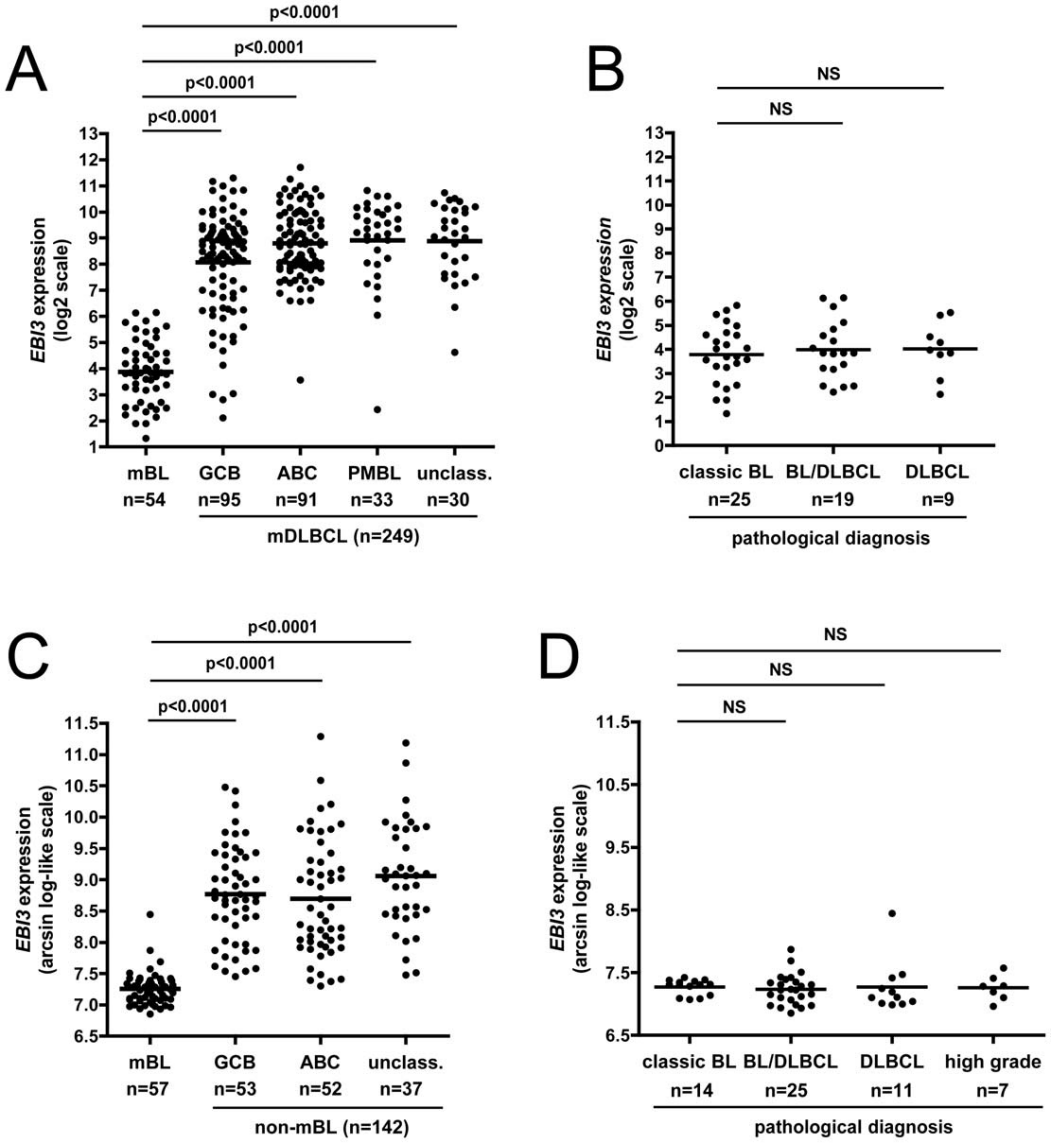
Differential expression profile of *EBI3* gene in BL and DLBCL defined by molecular profiling. Individual data for *EBI3* gene were retrieved from series GSE4732 [18] (A, B), and pooled from series GSE4475 and GSE10172 [8], [21] (C, D). Relative expression of *EBI3* is shown using the authors' scale. On (A) and (C) *EBI3* expression among the different molecular types defined by gene profiling is shown. On (B) and (D), the expression of *EBI3* in mBL is shown according to their original pathological diagnosis. p values are indicated. The horizontal bar indicates the mean. NS: not significant; unclass: unclassifiable.

First, we analyzed *EBI3* gene expression level in gene profiling studies performed in mature aggressive B-cell non-Hodgkin lymphomas that were available on Gene Expression Omnibus (GEO) of the National Center for Biotechnology Information. Individual data for *EBI3* were retrieved from the study from Dave *et al.* [18] (GSE4732) and from two German studies (Hummel *et al.* [8], GSE4475, and Klapper *et al.* [21], GSE10172). In these studies, cases of adult or pediatric lymphomas that had originally received a pathological diagnosis of BL, DLBCL, BL/DLBCL, or high grade mature B-cell lymphomas, unclassifiable, had been analyzed for gene expression and classified into molecular BL (mBL) or non-mBL (consisting mainly of DLBCL and designed as molecular DLBCL in ref [18], using classifiers that were specific to Dave *et al.* study or to the German studies. These 3 studies totaled 111 cases of mBL and 391 cases of non-mBL. These latter were further classified into GCB (n = 148), activated B-cell like (ABC) (n = 143) and PMBL (n = 33) subtypes, or left unclassified (n = 67). Data shown on Figure 1A and 1C indicated that *EBI3* expression significantly differed between mBL and each of the non-mBL subtype (p<0.0001). Thus, *EBI3* expression was low in all but one cases of mBL, whereas most cases of non-mBL, either GCB, ABC, PMBL, or unclassified, exhibited higher expression of *EBI3* gene. In Dave *et al.* study, cases of non-mBL of the GCB type were the most heterogenous for *EBI3* expression and contained the higher proportion of cases with low expression. However, this feature was not observed in the GCB non-mBL defined in the other studies, possibly due to the different ways the classification was performed in each study.

Over 50% of the cases - 28/53 (53%) (Figure 1B) and 43/57 (75%) (Figure 1D) -classified as mBL upon gene profiling by microarray analysis were initially classified as BL/DLBCL, DLBCL or aggressive NHL based on pathological diagnosis. Of note, *EBI3* level was equally low in classic BL and in cases that were re-classified as mBL upon molecular profiling (Figure 1B and 1D).

### Immunohistochemical analysis of EBI3 in BL and DLBCL

**Figure 2:**
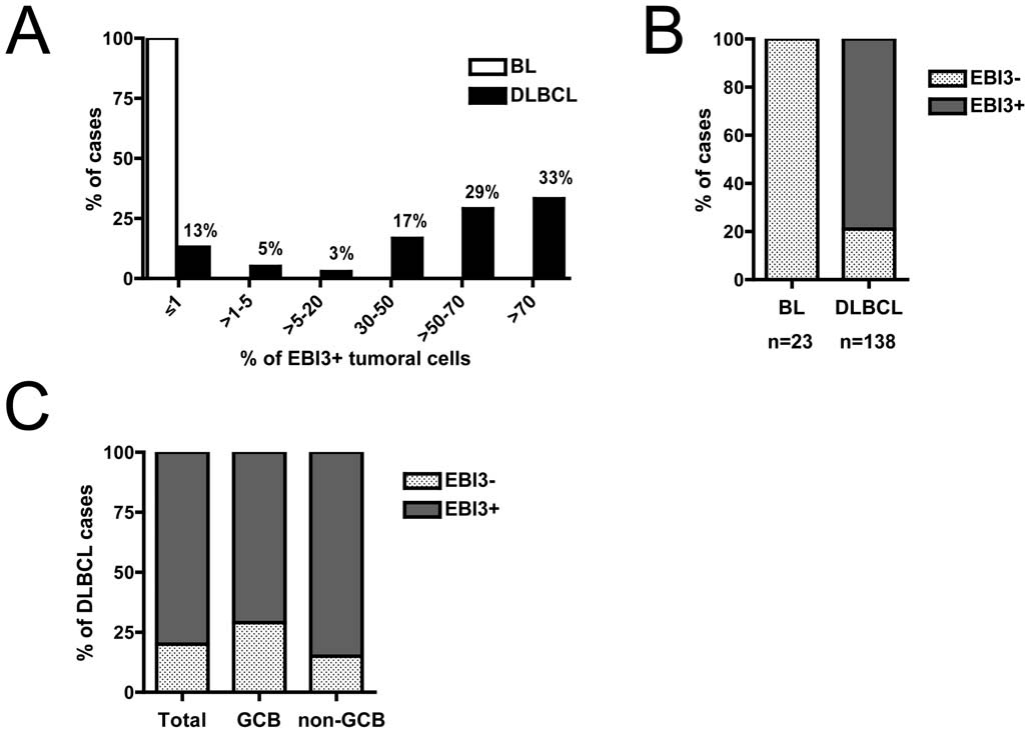
Immunohistochemical analysis of EBI3 expression in classic BL and DLBCL. (A) Percentage of EBI3-positive tumoral cells in BL and DLBCL cases analyzed by EBI3 immunohistochemistry. (B) Frequency of EBI3-positive or -negative cases among BL and DLBCL, when positivity is defined by ≥30% stained tumoral cells. (C) Distribution of EBI3-positive and -negative cases among DLBCL of the GCB or non-GCB subtypes.

The differential expression profile of *EBI3* observed among BL and DLBCL defined by molecular profiling was next confirmed at the protein level by analyzing by immunohistochemistry with anti-EBI3 mAb, 23 cases of classic BL and 138 cases of DLCBL (Figure 2A). No or rare EBI3-positive cells (1% positive tumoral cells at the most) were detected in BL cases including both EBV-positive or -negative cases. In contrast, numerous EBI3-positive cells, most of which were tumoral cells, were detected in a large proportion of DLBCL. These EBI3-positive tumoral cells exceeded 50% in over 60% of DLBCL cases. When a cut-off for positivity was set at ≥30% of tumoral cells (this cut-off will be used throughout the study), 109/138 (79%) of DLBCL cases were considered positive for EBI3, whereas all BL cases were negative (Figure 2B). When classified by immunohistochemistry in GCB and non-GCB subtypes, representing the equivalents of the GCB and ABC cell types defined by molecular profiling, 71% of the GCB cases were positive for EBI3, while this percentage reached 85% among non-GCB cases (Figure 2C), indicating a slight over-representation of EBI3-negative cases among GCB DLBCL, in line with the molecular data from Dave *et al.* study.

Taken together, these data obtained in a large series of BL and DLBCL indicate that positivity for EBI3 in tumoral cells strongly argues against a diagnosis of BL, while it is in favor of a DLBCL diagnosis.

### Lymphomas harboring *c-myc* translocations are characterized by lower expression of *EBI3* gene

**Figure 3:**
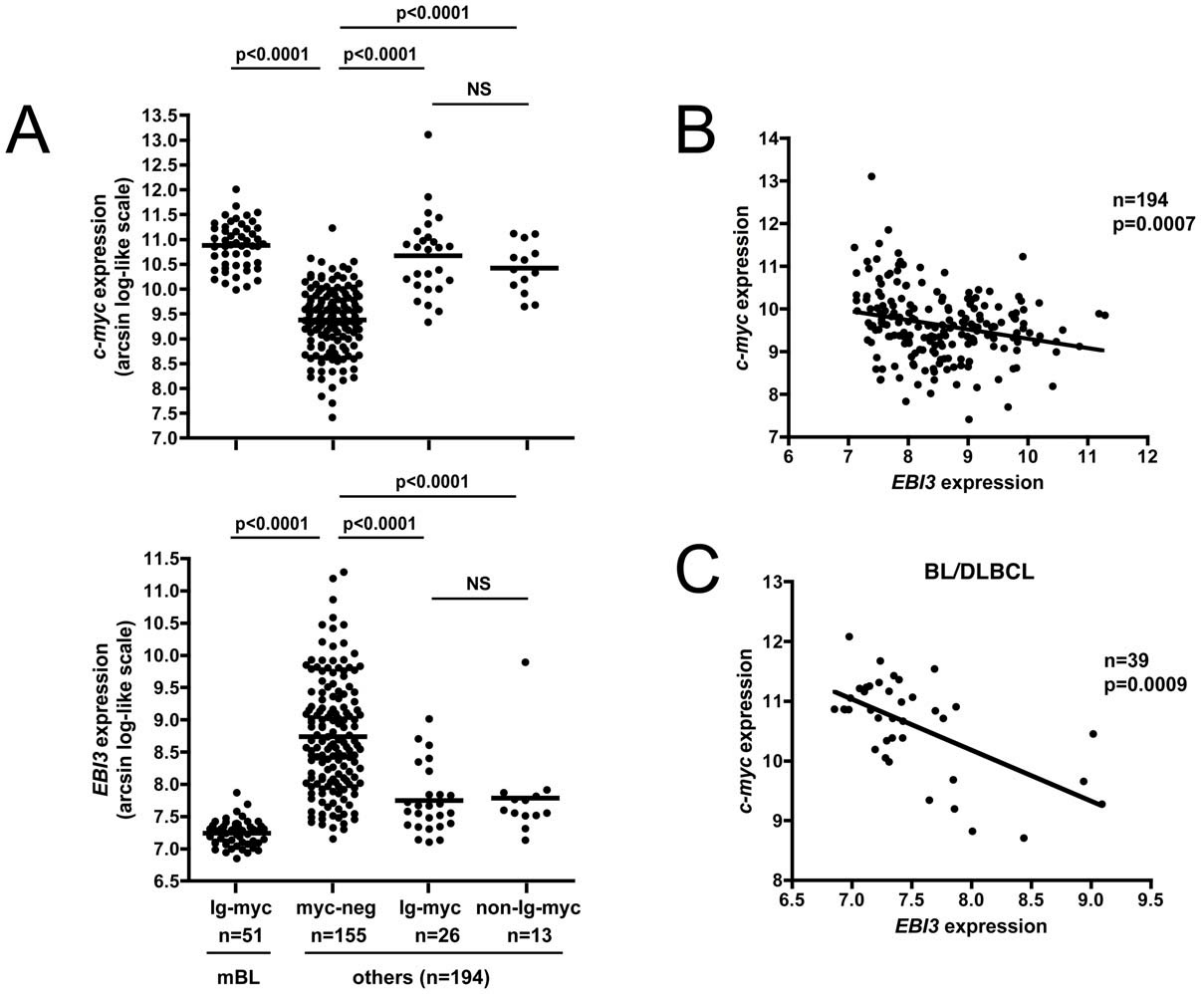
Analysis of *EBI3* gene expression according to the presence or absence of *c-myc* translocation. Individual data for *EBI3* and *c-myc* were retrieved from GSE4475 and GSE10172 series [8], [21]. On (A), the expression of *EBI3* and of *c-myc* was analyzed according to the presence or not of *c-myc* translocations involving an Ig locus (Ig-myc) or not (non-Ig-myc). « others » designated lymphomas other than mBL, ie lymphomas classifed as non-mBL and lymphomas that could not be classified as mBL or non-mBL. On (B), *EBI3* expression among all lymphomas other than mBL is represented according to that of *c-myc*. On (C), *EBI3* expression among lymphomas that had initially received a pathological diagnosis of BL/DLBCL is shown according to that of *c-myc*. p values are indicated. NS: not significant.

In the studies from Hummel *et al.* [8] and Klapper *et al.* [21], cytogenetic data for the *c-myc* gene were available for a large number of cases (n = 245). Therefore, we investigated in patients that did not fulfill the mBL signature, i.e. cases that were classified as non-mBL or cases that could not be classified into mBL or non-mBL and were called « intermediate forms », if the presence of *c-myc* gene rearrangements (21% of the cases) affected *EBI3* expression. Indeed, we observed that lymphomas, other than mBL, harboring *myc*-translocations had higher expression of *c-myc*, as expected, but also lower expression of *EBI3* (p<0.0001) (Figure 3A). This observation was true whether cases had *c-myc* translocations involving Ig or non-Ig genes. In these lymphomas, an inverse correlation between *EBI3* and *c-myc* expression was observed (Figure 3B)(n = 194, p = 0.0007). This inverse correlation was also observed when this analysis was performed by selecting cases that were originally classified as BL/DLBCL upon pathological diagnosis (Figure 3C)(n = 39, p = 0.0009).

Collectively, these data suggested that EBI3 expression level could be indicative of the level of c-myc expression and could constitute a marker to identify lymphomas with *c-myc* translocations, among both BL/DLBCL and DLBCL.

### Overexpression of c-myc represses *EBI3* gene expression

**Figure 4:**
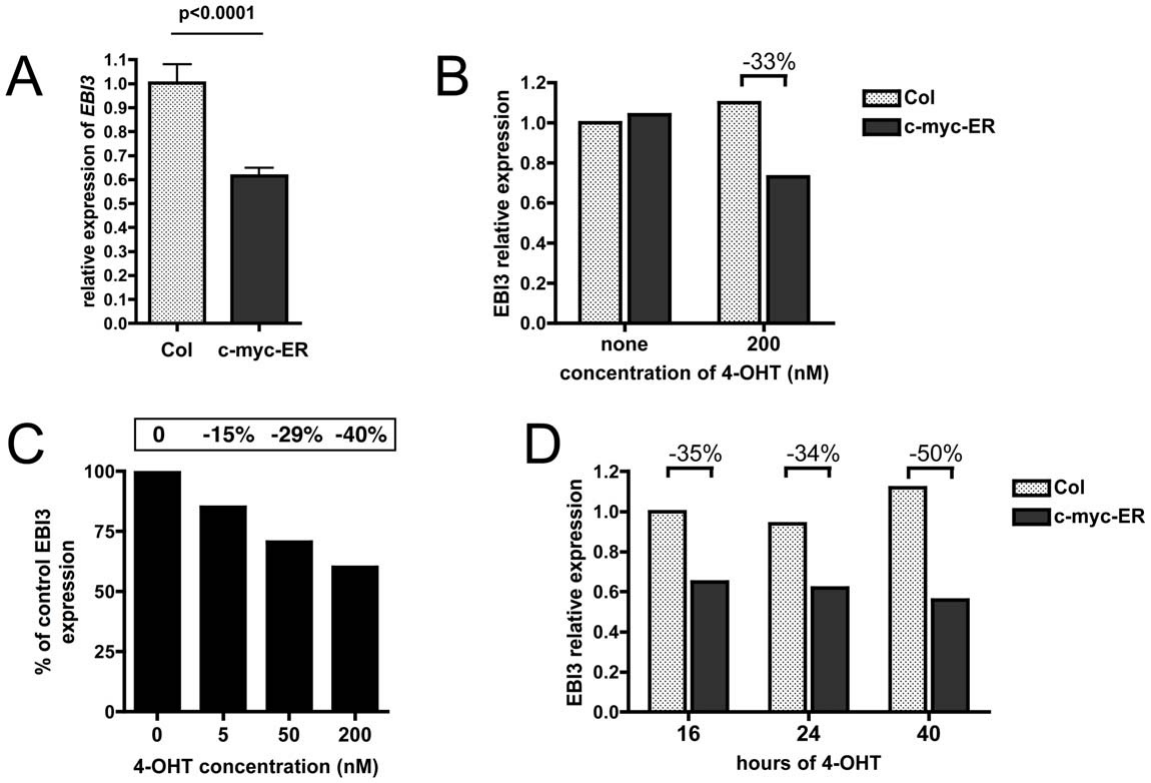
*In vitro* effect of c-myc overexpression on *EBI3* expression level. Karpas 1106 cell line was transfected with a GFP reporter plasmid together with c-myc-ER expression vector or a control vector and cultured for various times in the absence or presence of 4-OHT. *EBI3* expression in GFP-positive cells isolated from vector control transfected cells (Col) or c-myc-ER transfected cells (c-myc-ER) was analyzed by RTqPCR. In (A), transfected cells were cultured for 20 hours in the presence of 4-OHT (200 nM). Data are expressed as mean ± SEM from 3 independent transfections. p value is indicated. In (B), cells were cultured for 20 hours in the absence or presence of 4-OHT (200 nM). In (C), cells were cultured with increasing concentrations of 4-OHT. The expression of *EBI3* in c-myc-ER transfected cells relative to that measured in control tranfected cells is represented. The percentage of decrease is indicated. A higher concentration of 4-OHT (1 µM) did not result in higher repression of *EBI3* expression (not shown). In (D), 4-OHT was added to the transfected cells for the last 16, 24 or 40 h of the culture. In (B)–(D), a representative experiment is shown.

The inverse correlation observed between *EBI3* and *c-myc* expression suggested that c-myc overexpression may repress *EBI3* expression. To test this hypothesis, we used an *in vitro* assay by overexpressing an inducible c-myc-ER fusion protein that has been widely used to study c-myc regulated genes [22]. This fusion protein is retained in the cytoplasm as an inactive form and translocate to the nucleus when cells are exposed to a modified estrogenic ligand, 4-OHT. The Karpas 1106 DLBCL cell line, that expresses EBI3, was transiently transfected with a GFP reporter plasmid, and either pSG5 vector control or pSG5-c-myc-ER , and cultured in the presence of 4-OHT. Twenty hours post-transfection, GFP-positive cells were isolated by cell sorting and analyzed for *EBI3* expression by RTqPCR. Data shown on Figure 4A indicate that overexpression of c-myc results in statistically significant decrease of *EBI3* expression (40% decrease on average, p<0.0001). This effect was specific : it was not observed when 4-OHT was not added to the culture (Figure 4B) and was dose-dependent, as it gradually increased with 4-OHT concentration (Figure 4C). This negative effect of c-myc fusion protein overexpression on *EBI3* expression was observed at all time points tested upon 4-OHT addition (Figure 4D). Treatment of transfected cells with 4-OHT for 16 or 24 hours led to a similar decrease in EBI3 expression, while treatment for 40 h resulted in a somewhat higher decrease. Altogether, these data indicate that c-myc overexpression represses *EBI3* expression.

### Immunohistochemical analysis of EBI3 expression in BL/DLBCL and DLBCL characterized for *c-myc* gene rearrangement

Next, we investigated in DLBCL and BL/DLBCL whether their positivity or negativity for EBI3, assessed by immunohistochemistry, correlated with the absence or presence of *c-myc* translocation. This analysis was performed in 88 of the 138 cases of DLBCL tested for EBI3 for which cytogenetic data were available and in 23 cases of BL/DLBCL, all characterized for *c-myc* translocation, that we tested for EBI3 by immunohistochemistry. *c-myc* translocations were present in 12/88 (14%) of DLBCL cases and 19/23 (83%) of BL/DLBCL cases.

**Figure 5:**
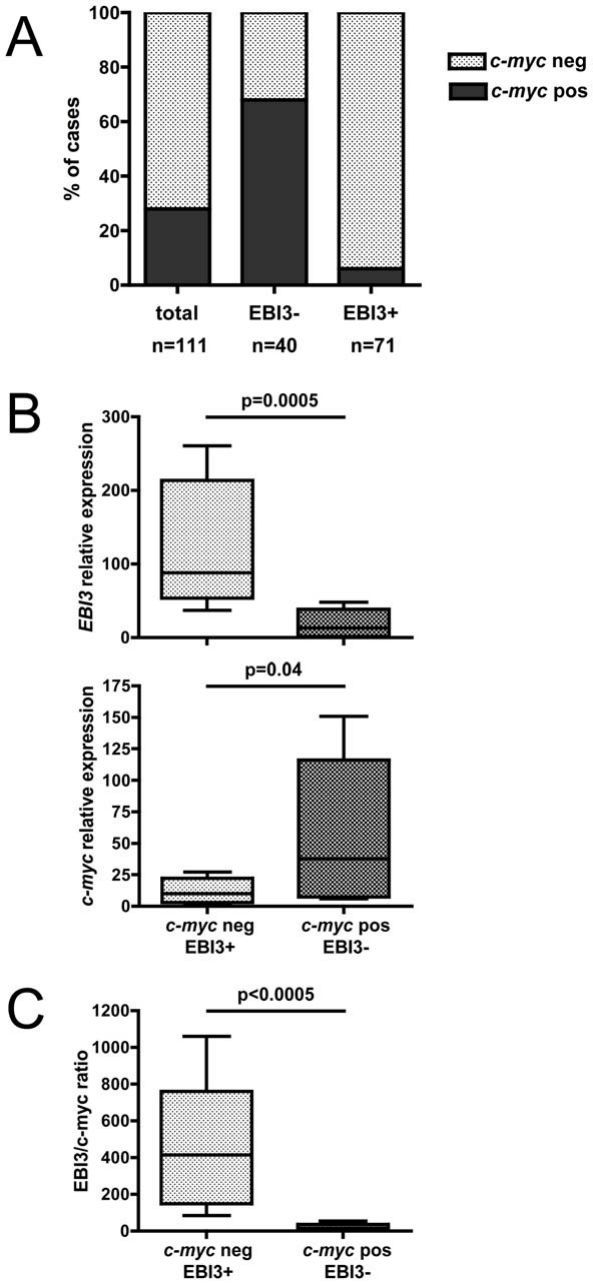
Effect of *c-myc* translocation on EBI3 expression in lymphomas. (A) The distribution of cases with or without *c-myc* translocations among all BL/DLBCL and DLBCL cases, or among EBI3-negative versus EBI3-positive cases, is shown. EBI3 positivity (≥30% stained tumoral cells) was assessed by immunohistochemistry. (B) The relative expression of *EBI3* and *c-myc* (normalized to *ß2m* expression) was determined by RTqPCR in frozen tissues from 10 cases of B-cell lymphomas without *c-myc* translocation that were positive for EBI3 by immunohistochemistry and 6 cases of B-cell lymphomas without *c-myc* translocation that were negative for EBI3. (C) The ratio of *EBI3/c-myc* expression in the cases analyzed is (B) is represented. p values are indicated.

**Figure 6:**
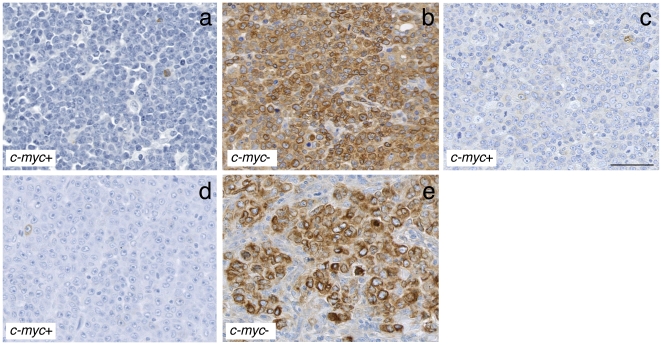
Immunohistochemical analysis of EBI3 expression in BL, BL/DLBCL and DLBCL. Sections from a case of BL (a), 2 cases of BL/DLBCL (b, c), and 2 cases of DLBCL (d, e) with or without *c-myc* translocation as indicated on each figure, were analyzed for EBI3 expression by immunohistochemistry. An inverse correlation between the presence of *c-myc* translocation and the positivity for EBI3 in tumoral cells was observed. Each picture is shown at the same magnification. The bar shown on (c) represents 50 µm.

Cases with *c-myc* translocation were not equally distributed among EBI3-positive or -negative cases (Figure 5A and Figure 6). Indeed, most EBI3-positive cases (67/71, 94%) did not have *c-myc* translocation. In contrast, while 28% of total cases had a *c-myc* translocation, this percentage reached 68% among EBI3-negative cases. The correlation between negativity for EBI3 and the presence of *c-myc* gene rearrangement was higher in BL/DLBCL than in DLBCL. Indeed, 16/17 (94%) of EBI3-negative BL/DLBCL had a translocation for *c-myc*, while 12/23 (52%) of EBI3-negative DLBCL had *c-myc* gene rearrangement. Nevertheless, the frequency of *c-myc* translocation was 3.7 higher in EBI3-negative DLBCL cases compared to all DLBCL cases. Thus, consistent with the previous observations, positivity for EBI3, as determined by immunohistochemistry, argues against the presence of *c-myc* translocation. Conversely, negativity for EBI3 among BL/DLBCL or DLBCL indicates a higher probability for the presence of *c-myc* translocation, especially in BL/DLBCL cases.

Frozen tissue was available in 16 of the 88 cases with cytogenetic data. These cases included 10 cases without *c-myc* translocation (all DLBCL cases) that contained from 50→90% EBI3-positive tumoral cells as determined by immunohistochemistry, and 6 cases with *c-myc* translocation (5 BL/DLBCL cases and one DLBCL case) that contained <5% EBI3-positive tumoral cells. RTqPCR analysis confirmed that cases with *c-myc* translocation, expressing higher levels of *c-myc* (Figure 5B, bottom graph, p = 0.04) and classified as negative by EBI3 immunohistochemistry, had low *EBI3* gene expression compared to cases without *c-myc* translocations classified as EBI3-positive (Figure 5B, top graph, p = 0.005). On average, the *EBI3/c-myc* expression ratio was 27-fold higher in lymphomas without *c-myc* translocations than in lymphomas with *c-myc* translocation (Figure 5C, p<0.005). These data indicate that EBI3 expression, analyzed at the protein level by immunohistochemistry or at the mRNA level, inversely correlates with *c-myc* expression.

### In vivo downregulation of EBI3 expression by tumoral cells upon *c-myc* translocation

**Figure 7:**
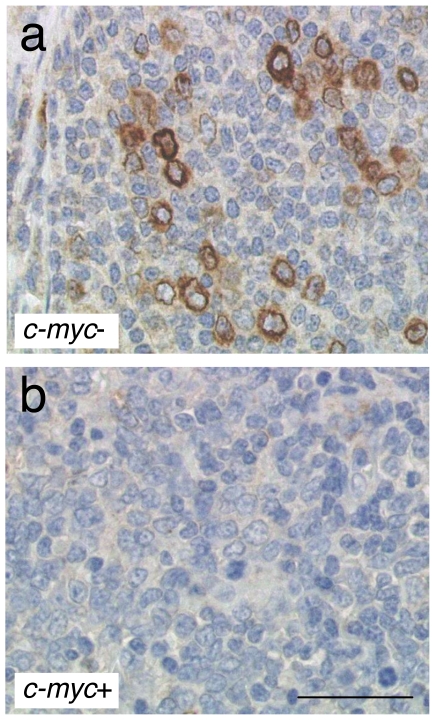
Immunohistochemical analysis in a case of follicular lymphoma and its transformed counterpart. Sections from a follicular lymphoma (a) that subsequently acquired a *c-myc* translocation and transformed into a DLBCL (b) were analyzed for EBI3 expression by immunohistochemistry. The bar represents 50 µm.

*C-myc* translocations can occur as secondary oncogenic event in follicular lymphomas that transform into DLBCL. In a previous report, we had observed that in follicular lymphomas, EBI3 was expressed by tumoral cells, mainly large cells, at a variable level that could reach up to 30% of tumoral cells [15]. To further investigate the *in vivo* effect of *c-myc* translocation on EBI3 expression, we analyzed the expression of EBI3 by immunohistochemistry in a case of follicular lymphoma that had acquired *c-myc* translocation during transformation (Figure 7). Whereas a substantial fraction of tumoral cells stained positive for EBI3 in the follicular lymphoma (Figure 7a), tumoral cells were all negative for EBI3 in the *c-myc*-positive transformed counterpart (Figure 7b). This *in vivo* observation further supports our *in vitro* data showing that c-myc overexpression downregulates EBI3 expression.

## Discussion

DLBCL is a heterogeneous lymphoma with various subtypes characterized by different gene expression profiles [23]–[25], which hampers the identification of markers that are both specific to DLBCL and common to all subtypes. The analysis of data from gene profiling studies available on GEO, and our data obtained by immunohistochemistry, all concurr to establish that DLBCL and BL are characterized by a differential expression profile of EBI3. In particular, our immunohistochemical analysis indicates that, whereas EBI3 was not expressed at significant levels in all BL cases, a large fraction of tumoral cells was positive for EBI3 in ∼80% of DLBCL cases. In addition, we showed that an inverse correlation was observed between EBI3 expression and the presence of a *c-myc* translocation. Thus, 94% EBI3-negative BL/DLBCL cases exhibited *c-myc* translocations. In addition, while *c-myc* translocations were found in 14% of DLBCL cases in our series, this percentage increased to 52% among EBI3-negative cases. As mentioned earlier, the identification of *c-myc* translocations among BL/DLBCL and DLBCL is important, given that cases with *c-myc* translocations are associated with poor prognosis and decreased survival [8], [9], [11], [12]. Despite a recent report suggesting that *c-myc* translocation could be identified by analyzing c-myc subcellular localization [26], the overexpression of c-myc resulting from the translocation of the gene remains difficult to assess by immunohistochemistry. Thus, EBI3 immunohistochemistry, possibly in conjunction with c-myc staining, performed routinely in all cases of BL, DL/DLBCL and DLBCL, could not only help to discriminate BL and DLBCL, but also be useful to identify cases of BL/DLBCL and DLBCL with potential *c-myc* translocation and target these cases for further cytogenetic analysis by FISH. Because of practical considerations, FISH analysis is usually not routinely performed in all cases of DLBCL. Given that *c-myc* translocations are mostly found among EBI3-negative DLBCL cases which account for about one fifth of all DLBCL cases, targeting EBI3-negative cases for FISH analysis would allow to reduce by 80% the total number of DLBCL to test.

The factors regulating EBI3 expression in B-cell lymphomas remain to be established. In normal B cells, EBI3 is expressed at precise stages of B-cell differentiation. It is not expressed in naive B cells and in centroblasts, and is essentially expressed by a subset of germinal center B cells corresponding to activated centrocytes or cells at an early stage of plasma cell differentiation [15], [27]. In activated normal B cells, EBI3 expression is positively regulated by NF-κB activation [15]. In tumoral B cell lines, including DLBCL cell lines, EBI3 expression has been shown to be dependent on NF-κB activation, and in EBV-transformed B cells to be induced by LMP1 in an NF-κB dependent manner [14], [16], [28]. Thus, the absence of EBI3 expression in BL may be due to its stage of differentiation (centroblast), its lack of NF-κB activation, the absence of LMP1 expression (for EBV-positive cases) or its high expression of c-myc that could repress EBI3 induction. Of note, the only mBL showing significant expression of *EBI3* (Figure 1C) was a case that did not exhibit *c-myc* translocation.

In DLBCL, both its stage of differentiation and the activation of NF-κB, may account for EBI3 expression. Previous studies have shown that DLBCL originates from GC (GCB subtype) or post-GC (ABC subtype) normal B cells [23]. The GCB subtype was initially associated with low NF-κB activation, whereas the ABC subtype was associated with high NF-κB activation [23]. However, a more recent study has shown that not only 95% of ABC forms, but also nearly 50% of GCB forms, are characterized by substantial NF-κB activation [29]. Thus, in both GCB and ABC subtypes, the activation of NF-κB may account for EBI3 expression. The higher proportion of GCB cases among EBI3-negative cases supports this hypothesis.

Another factor regulating EBI3 expression in DLBCL is the presence of *c-myc* translocations resulting in c-myc overexpression that in turn can repress EBI3 expression. Indeed, *c-myc* translocations were present in about half EBI3-negative DLBCL cases. This negative effect of c-myc overexpression on EBI3 expression may be a direct effect or could result from the known negative effect of c-myc on NF-κB effectors [30], [31]. The negativity for EBI3 expression among DLBCL cases may also be due to the fact that some of them may arise from transformed follicular lymphomas, an event that is not always diagnosed. Previously, we have shown that EBI3 expression by tumoral cells is lower in follicular lymphomas (from 1 to 30% positive tumoral cells at the most) than in DLBCL [15], and remains lower (less than 30% positive tumoral cells) in established transformed follicular lymphomas (our unpublished data). This hypothesis is also supported by the fact that *EBI3* gene expression in the German series analyzed here [8], [21] was on average lower in DLBCL with t(14;18) translocation (not shown).

The biological significance of EBI3 expression in DLBCL remains to be investigated. EBI3 can associate with two different partners, p28, to form the heterodimeric cytokine IL-27, or p35, to form IL-35 [32], [33]. Both cytokines have been described as important negative regulators of T-cell responses [34]–[36]. In a previous immunohistochemical analysis, p28 was not detected in tumoral cells of DLBCL cases [15]. *p28*, the expression of which was analyzed only in a subset of the lymphomas of the gene profiling studies studied here, did not show increased expression in DLBCL compared to BL. *p35* gene was present on the gene chips used in the 3 studies, and analysis of its expression profile showed increased expression in DLBCL, especially those of the ABC subtype, compared to BL (our unpublished data). Whether IL-35 protein is produced in these lymphomas and contributes to their higher aggressiveness possibly by suppressing local anti-tumoral immune responses, remains to be established.
